# WDHD1 facilitates G1 checkpoint abrogation in HPV E7 expressing cells by modulating GCN5

**DOI:** 10.1186/s12885-020-07287-1

**Published:** 2020-09-03

**Authors:** Yunying Zhou, Fengyan Pei, Mingyu Ji, Fang Zhang, Yingshuo Sun, Qianqian Zhao, Xiao Wang, Yatian Hong, Juanjuan Tian, Yunshan Wang, Jason J. Chen

**Affiliations:** 1 Medical Research & Laboratory Diagnostic Center, Jinan Central Hospital, Cheeloo College of Medicine, Shandong University, Jinan, Shandong China; 2 Department of Microbiology, School of Basic Medical Sciences, Cheeloo College of Medicine, Shandong University, Jinan, 250012 Shandong China; 3 Microbiology Department, Jinan Central Hospital Affiliated to Shandong first medical university, Jinan, China; 4 Shandong LaiBo Biotechnology co., Ltd, Jinan, China

**Keywords:** WDHD1, GCN5, P-Akt, G1 arrest, HPV

## Abstract

**Background:**

Genomic instability is a hallmark of cancer. The G1 checkpoint allows cells to repair damaged DNA that may lead to genomic instability. The high-risk human papillomavirus (HPV) E7 gene can abrogate the G1 checkpoint, yet the mechanism is still not fully understood. Our recent study showed that WDHD1 (WD repeat and high mobility group [HMG]-box DNA-binding protein 1) plays a role in regulating G1 checkpoint of E7 expressing cells. In this study, we explored the mechanism by which WDHD1 regulates G1 checkpoint in HPV E7 expressing cells.

**Methods:**

NIKS and RPE1 derived cell lines were used. Real-time PCR, Rescue experiment, FACS and BrdU labeling experiments were performed to examine role of GCN5 in G1 checkpoint abrogation in HPV-16 E7 expressing cells.

**Results:**

In this study, we observed that WDHD1 facilitates G1 checkpoint abrogation by modulating GCN5 in HPV E7 expressing cells. Notably, depletion of WDHD1 caused G1 arrest while overexpression of GCN5 rescued the inhibitory effects of WDHD1 knockdown on G1/S progression. Furthermore, siWDHD1 significantly decreased cell cycle proliferation and DNA synthesis that was correlated with Akt phosphorylation (p-Akt), which was reversed by GCN5 overexpression in HPV E7 expressing cells.

**Conclusions:**

In summary, our data identified a WDHD1/GCN5/Akt pathway leading to the abrogation of G1 checkpoint in the presence of damaged DNA, which may cause genomic instability and eventually HPV induced tumorigenesis.

## Background

The human papillomaviruses (HPVs) are spherical small DNA viruses that induce lesions in the skin and mucosa. The high-risk (HR) HPVs infection may lead to cervical cancer and other cancers. Up to 75% of cervical cancers are caused by HPV genotypes 16 and 18 [1]. The transforming properties of HR HPVs mainly depend on E6 and E7 oncogenes [2], which inactivate p53 and Rb family members respectively, thus abrogating cell cycle checkpoints [3]. HR HPV E7 can promote pRB degradation, which result in release of transcription factor E2F, transcription of genes required for DNA replication, and cell proliferation disorder [4–8].

The cell cycle progression is modulated at cell-cycle checkpoints by multiple factors such as cyclins, cyclin-dependent kinases (Cdks) [9]. Once the checkpoint becomes abnormal, genomic instability may occur [10]. Genomic instability is a hallmark of cancer progression [10], and G1 checkpoint determines whether cells can enter S phase for DNA replication. In the early G1 phase, pRb is partially phosphorylated of by Cdk4-Cdk6. pRb is completely phosphorylated by Cdk2 in the late G1 phase.

Genomic DNA of the normal cells duplicates only once per cell cycle. Replication starts in two steps: assembling and activating of pre-replication (pre-RC). The assembly of the pre-RC complex is regulated by cell cycle, which mainly occurs in the late mitosis and G1 phase. Prior to S phase, origins are licensed by the binding of components of the replicative DNA helicase in eukaryotes. Recognition complex (ORC), Cdc6, Cdt1 and MCM2–7 was recruited successively to the DNA replication starting point to participate in the assembly of the pre-RC complex and then initiate DNA replication [11]. It is generally believed that the DNA replication initiation factor affects the G1 checkpoint by regulating the initiation of DNA replication, which in turn causes G1 arrest. WDHD1 (WD repeat and HMG - box DNA - binding protein 1) was also shown to be involved in the assembly process [12]. In addition, WDHD1 act as a G1 checkpoint control protein [13–15]. How WDHD1 precisely regulates G1 checkpoint remains to be illucidated.

We have demonstrated a role for WDHD1 in G1 checkpoint control in HPV E7 expressing cells [16]. Our result suggests that WDHD1 may regulates G1 checkpoint through a mechanism independent of DNA replication initiation. This study aims to understand how WDHD1 regulates G1 checkpoint in E7 expression cells. It was reported that GCN5(histone acetyltransferase complex) plays a role in the G1 checkpoint control while WDHD1 inhibits degradation by disrupting its interaction with ubiquitination ligase CRL4 complex [17, 18]. Our recent study revealed that GCN5 promotes HPV expressing cell proliferation by regulating E2F1 [19]. In the present study, we determined the role of GCN5 and the mechanism by which WDHD1 abrogates G1 checkpoint in E7 expressing cells.

## Methods

### Cell culture

pBabe retroviral system was used to establish the HPV-16 E7 expressing NIKS and RPE1 cells as described previously [20]. Puromycin was used to maintain the above two cell lines, which are limited to be used within 15 generations [16].

### RNA-seq

NIKS cells were used to extract total for construct cDNA libraries construction. The detailed operation processes please refer to the RNA-seq section of our published article [16].

### Real-time PCR

According to the kit’s instructions, the entire RNA was separated with Qiagen RNeasy kit from the E7 expressing RPE1 or NIKS cells and their correspondent vector-control cells. Invitrogen cDNA synthesis kit and Bio-Rad SYBR Green Supermix were used for synthesis cDNA and Real-Time PCR. Details need to refer to the published articles [6, 16].

### Flow cytometry

BrdU (bromodeoxyuridine) labeling and cell cycle experiments were performed on BD FACSAria™ III sorter equipment and analyzed by Cytomics™ FC500 Flow Cytometry CXP 2.0. The concentration of the Alexis Biochemicals bleomycin used in the experiment was 4 μg/ml. The specific experimental steps need to follow the published articles [16, 19, 20].

### siRNAs and transfection

The Invitrogen Lipofectamine 2000 transfection reagent was used for gene knockdown and cell cycle analysis in E7 and vector-control expressing cells. The sequence of siRNA duplexes were in Table 1. Detailed experimental steps need to follow our published article [16].

**Table 1:**
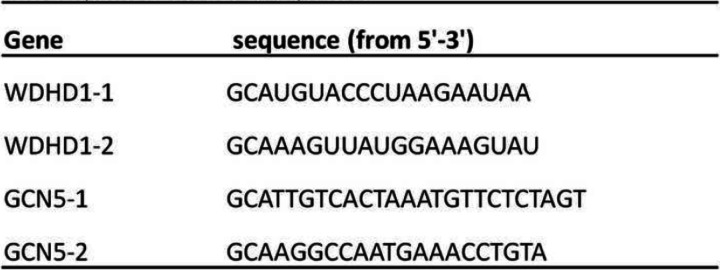
The sequence of siRNA duplexes

### Immunoblotting

Protein extracted from cells were measured by Pierce BCA (bicinchoninic acid), and then proceeded to SDS PAGE (polyacrylamide gel) electrophoresis, finally to be detected with antibodies against WDHD1 (abcam, ab72436), GCN5 (Santa Cruz, Sc-365,321), and tubulin (Sigma, T-4026), AKT (CST, 4685), p-AKT (CST, 4060). Detailed experimental steps need to follow our published article [16]. The Half Life Calculator was used to calculate the half-life of GCN5 which was treated with 25 μg/ml CHX (cycloheximide) (www.calculator.net).

### Statistical analysis

Means and standard deviations (SDs) were used to present the data and the differences between means were compared by the student’s t-test. *P* < 0.05 was considered significant.

## Results

### Expression of GCN5 correlates with WDHD1 in HPV-16 E7 expressing cells

**Fig. 1:**
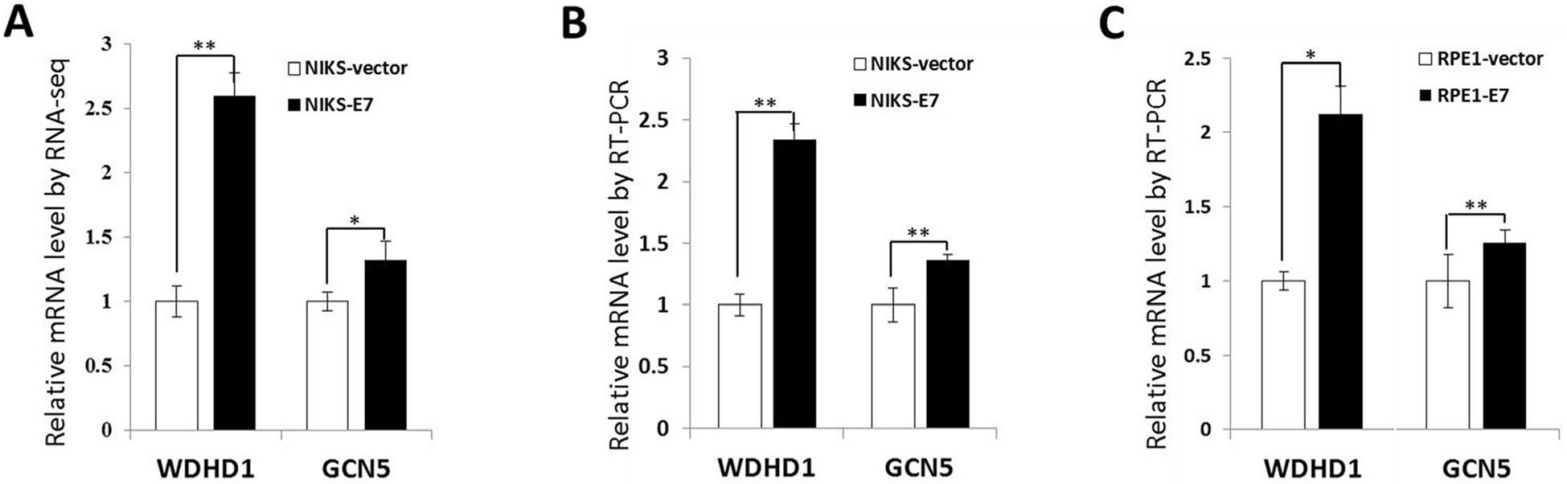
GCN5 mRNA expression correlates with WDHD1. **a** GCN5 and WDHD1 mRNA levels in NIKs cells determined by RNA-seq. **b** and **c** GCN5 and WDHD1 mRNA levels in NIKS and RPE1 cells determined by real-time PCR. Error bars reflect the standard deviations of the mean. *, *p* < 0.05 **, *p* < 0.01

Our previous study demonstrated a role for WDHD1 in G1 checkpoint abrogation E7 expressing cells [16]. It was reported that GCN5 plays a role in the G1 checkpoint control while WDHD1 inhibits its degradation [17–19]. Moreover, our recent study revealed that GCN5 also promotes cell cycle progression in HPV E7 expressing cells [19]. We therefor hypothesize that WDHD1 performs its cell cycle promting function by up-regulating GCN5 in E7 expressing cells. We noticed that both WDHD1 and GCN5 expression were elevated in our RNA-seq data in HPV E7 expressing NIKs cells (Fig. 1a) [18]. This was confirmed by an RT-PCR analysis in both NIKS cells (Fig. 1b) and RPE1 cells (Fig. 1c).

**Fig. 2:**
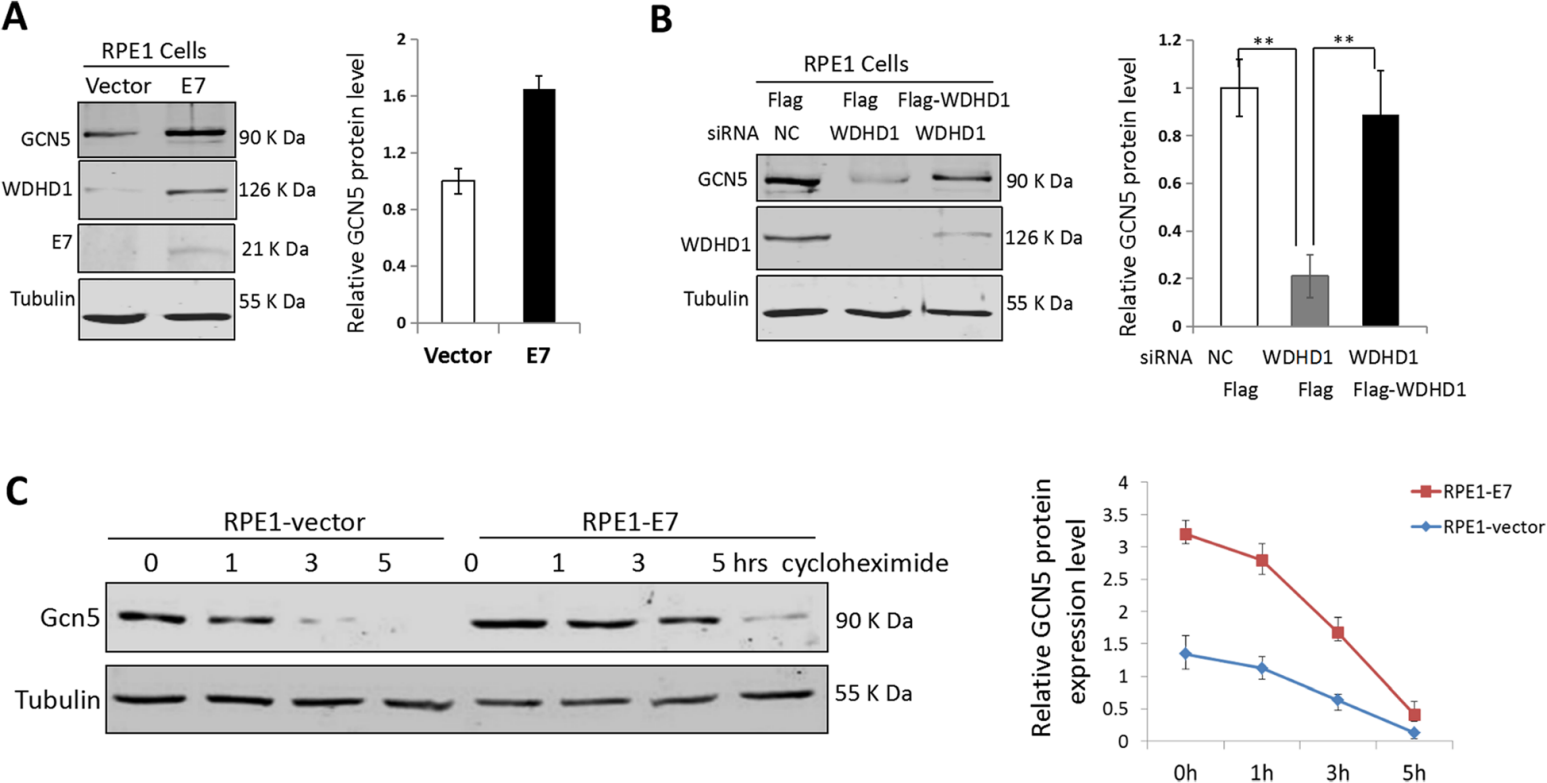
GCN5 protein expression correlates with WDHD1. **a** GCN5 and WDHD1 protein levels in RPE1 cells. **b** GCN5 protein levels in RPE1 E7 cells examined by Western blotting after retransfected with Flag-WDHD1. Data were summarized (Right panel). **c** RPE1 cells were incubated with cycloheximide (CHX) and harvested at the indicated times. The stability of GCN5 was monitored by immunoblotting analyses (left panel). Data are summarized in the right panel. Data from a representative experiment of 3 are shown, *P < 0.05; **P < 0.01. The WB samples for quantitative comparisons on different gels/blots derive from the same experiment and that gels/blots were processed in parallel. Cropping is used for the gels and blots in the main paper and the ‘full-length blots/gels are presented in Supplementary Figure 2

Furthermore, the steady-state levels of WDHD1 and GCN5 were also increased in E7 expressing RPE1 cells (Fig. 2a). The increased steady-state level of GCN5 was regulated by WDHD1, as siRNA knockdown of WDHD1 reduced GCN5 while this reduction was reveres after transfection of cells with WDHD1 (Fig. 2b). We also determined the stability of GCN5 protein in E7 expressing cells. Accordingly, after treatment with cycloheximide, the steady-state levels of GCN5 in E7 expressing and vector control cells were measured (Fig. 2c). The results indicated that the half-life of GCN5 protein in E7 expression cells was significantly higher than that in the control cells (3.8 h versus 1.4 h).

### Overexpression of GCN5 overrides G1 checkpoint activation and S-phase entry delay induced by WDHD1 knock-down

**Fig. 3:**
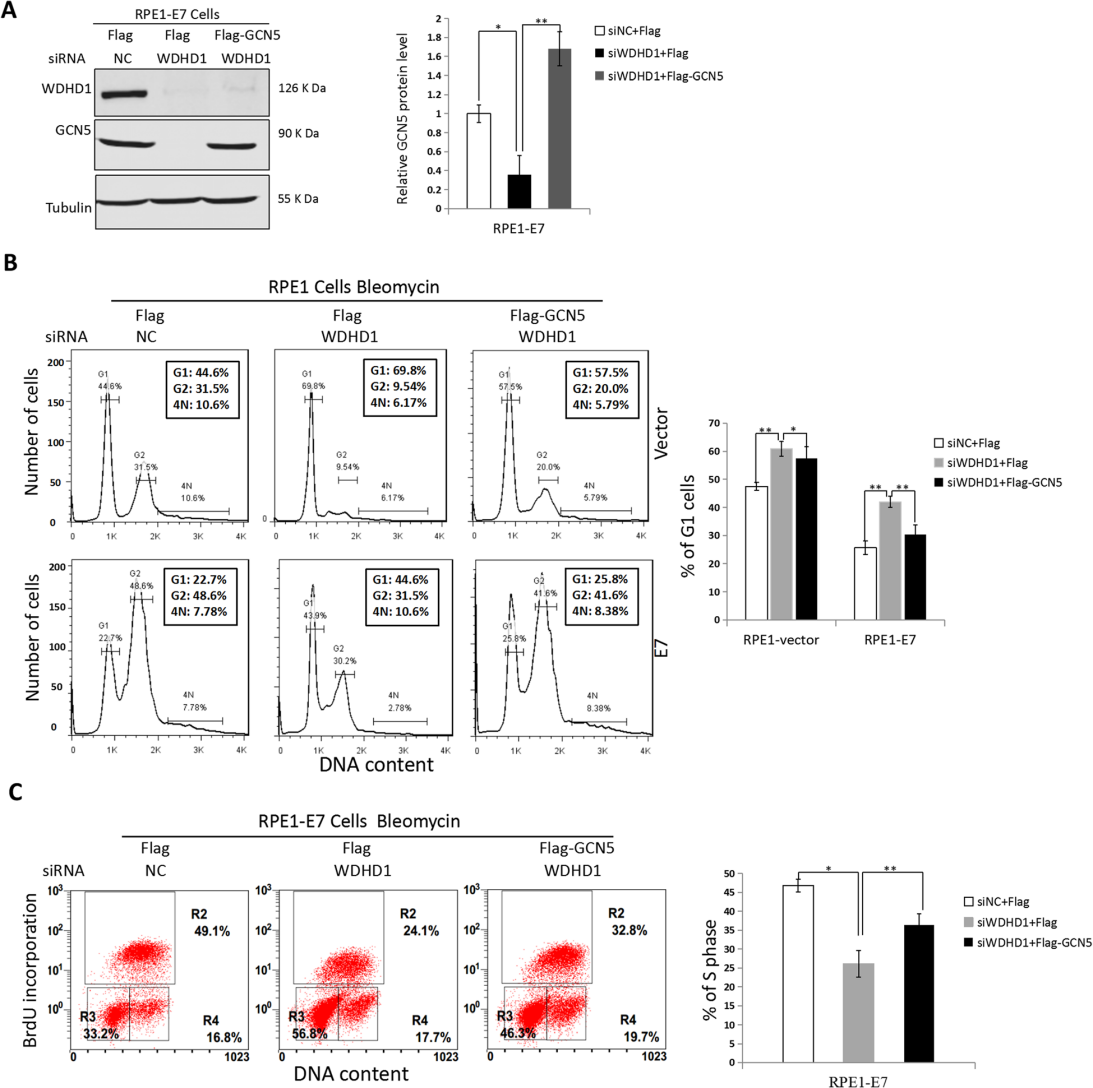
Over expression of GCN5 rescues DNA replication reduction induced by WDHD1 depletion. **a** RPE1 cells expressing E7 were transfected with Flag-GCN5 or flag after WDHD1 siRNA transfection, and then cells treated with bleomycin for 36 h. Western blotting was used to determine the steady-state level of GCN5. Tubulin was used as a loading control. Right panel, quantification of relative GCN5 levels from 3 independent experiments. The WB samples for quantitative comparisons on different gels/blots derive from the same experiment and that gels/blots were processed in parallel. Cropping is used for the gels and blots in the main paper and the ‘full-length blots/gels are presented in Supplementary Figure 3A. **b** Cells were stained with PI after bleomycin treatment. G1, S and G2 phases are indicated and quantified. **c** Cells were stained with BrdU after bleomycin treatment, and analyzed by flow cytometry. Data from a representative experiment of 3 were shown. Error bars reflect the standard deviations of the mean. NC, negative control. *P < 0.05; **P < 0.01

We have demonstrated that both WDHD1 and GCN5 play key roles in S-phase entry and G1 checkpoint control in E7 expressing cells [16, 19], and our data suggest that WDHD1 regulates GCN5 expression (Figs. 1 and 2). To establish the functional interaction of the two molecules, we performed a rescue experiment. For this goal, we demonstrated that transfection of GCN5 can restore the steady-state levels of GCN5 protein caused by WDHD1 siRNA interference (Fig. 3a). Significantly, the percentage of cells arrested at the G1 phase as a result of WDHD1 knockdown was reduced after GCN5 expression (from 43.4 to 31.1%) (Fig. 3b). Similarly, percentage of BrdU incorporation due to WDHD1 knockdown was increased (from 24.3 to 31.6%) after GCN5 expression (Fig. 3c). These results demonstrated that the G1 abrogation and S-phase entry caused by WDHD1 knocking down could be rescued by up-regulating GCN5 in HPV E7 expressing cells.

### WDHD1 activates Akt via GCN5 in E7 expressing cells

**Fig. 4:**
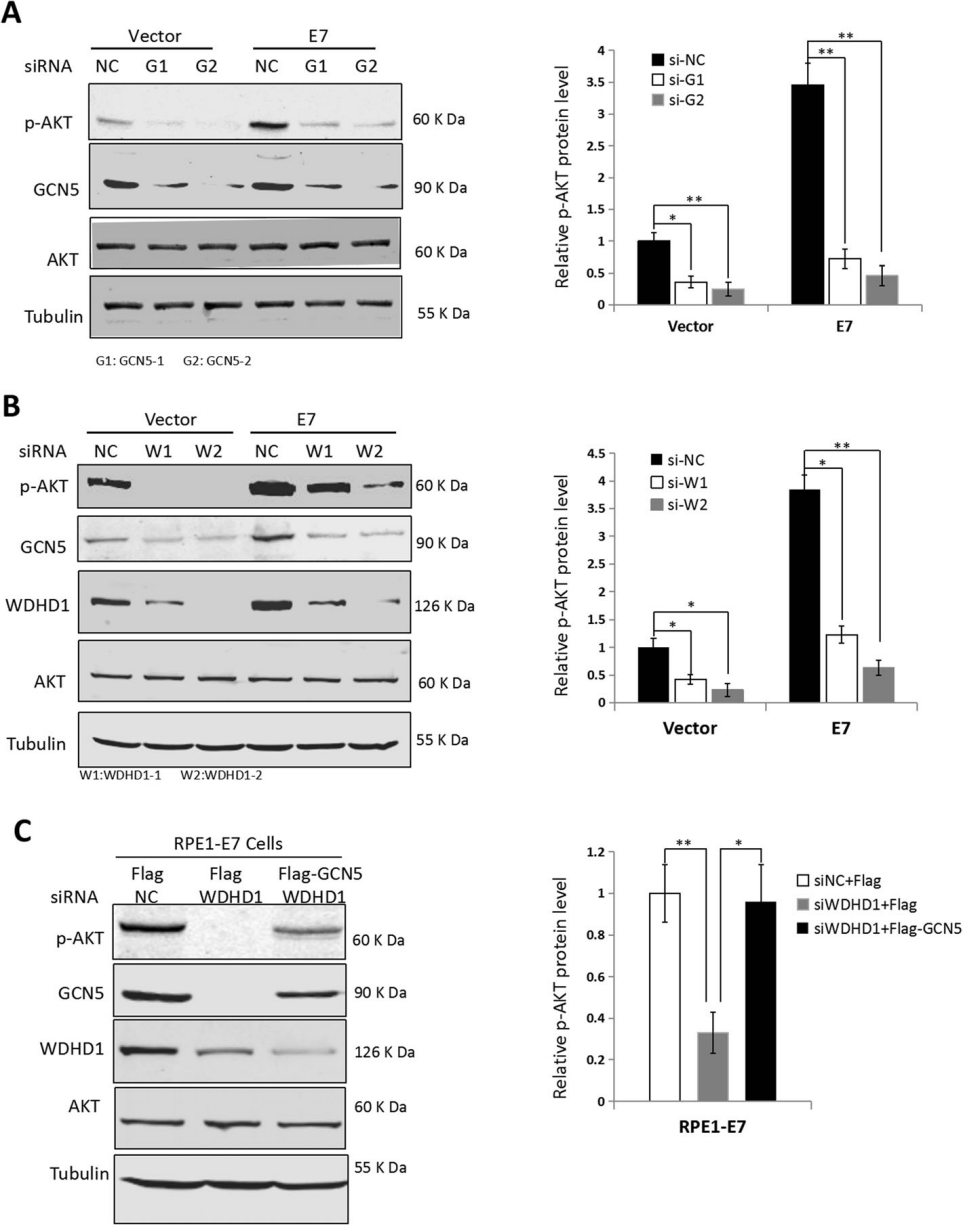
WDHD1 activates p-Akt expression to modulate cell cycle progression by up-regulating GCN5 in HPV E7 expressing cells. **a** The steady state levels of p-Akt by Western blot after GCN5 interference in RPE1 cells. **b** The steady state levels of p-Akt and GCN5 by Western blot after WDHD1 interference in RPE1 cells. **c** RPE1-E7 expressing cells were transfected with Flag-GCN5 or flag after WDHD1 siRNA transfection. The steady-state levels of p-Akt and GCN5 were measured by Western blotting. Tubulin was used as a loading control. Right panel, quantification of relative p-Akt levels from 3 independent experiments. Error bars reflect the standard deviations of the mean. NC, negative control. *P < 0.05; **P < 0.01. The WB samples for quantitative comparisons on different gels/blots derive from the same experiment and that gels/blots were processed in parallel. Cropping is used for the gels and blots in the main paper and the ‘full-length blots/gels are presented in Supplementary Figure 4

It was reported that GCN5 regulates the G1 checkpoint by activating Akt [21]. As shown in Fig. 4a, activation of Akt, as indicated by its phosphorylation at 473, decreased after GCN5 knockdown, indicating that GCN5 regulates AKT activity in E7 expressing cells. We then examined whether WDHD1 could activate Akt by modulating GCN5, and found that GCN5 and p-Akt levels were greatly decreased in E7 expressing cells when WDHD1 were knocked down (Fig. 4b).

We have already showed that expression of GCN5 can rescue G1 arrest caused by WDHD1 knockdown, we then asked whether expression of GCN5 can compensate for the inactivation of Akt caused by WDHD1 knockdown. As shown in Fig. 4c, expression of GCN5 can indeed compensate for Akt inactivation caused by WDHD1 knockdown. Therefore, taking all the results into consideration, cell cycle progression could be modulated by WDHD1 activating of Akt via GCN5 up-regulation in HPV E7 expressing cells.

## Discussion

Licensing checkpoints including the DNA replication initiation factors regulate the S-phase entry [11, 21]. Previously, WDHD1 was thought to have a role in pre-RC assembly [13, 16]. However, the biochemical mechanism has not been verified at the cellular level in cervical cancer. Notably, we have demonstrated that even WDHD1 partially knocked down to control cells level can still induces G1 arrest [16], suggesting that besides the replication initiation mechanism, WDHD1 may regulate the G1 checkpoint through other mechanism or other target gene. In addition, WDHD1 is considered to be required for the stability of histone acetyltransferase GCN5 [18], the latter was reported to promote G1/S phase transition and cell cycle progression [22–24] by affecting its downstream target genes such as HBXIP [23], EGR2 [25], AIB1 [26], c-Myc [27, 28], E2F1 [22]. In addition to our recent results showing that GCN5 upregulates E2F1 and thus promotes HPV E7 induced cell proliferation [19], the other roles of GCN5 in cervical cancer are still unclear. This current study elucidates a model that HPV E7 up-regulates the expression of WDHD1 and then GCN5, which activates Akt and thereby promotes cell proliferation.

Due to the key regulatory role in multiple cell processes including cell proliferation, differentiation and apoptosis, AKT (also known as PKB) has received much attention in the field of life sciences. AKT signaling is typically activated in invasive squamous cell carcinomas [29, 30], including nearly 80% of cervical cancers [31–33]. However, the function of GCN5 activates AKT in cervical cancer has not been reported. Previous studies have shown that HPV E6 and E7 oncogenes augment the activation of AKT [32, 34]. Nonetheless, a recent study showed that HPV-16 E7 can attenuate pAKT signalling [35]. In addition, hypoxic AKT activation is observed under conditions of E6/E7 repression [36], which indicates that the hypoxic AKT activation is regulated by an E6/E7-independent mechanism, but the mechanism is unclear. These suggest that several pathways exist in activation of AKT signaling in HPV-positive cervical cancers. In this study, GCN5 was further discovered to modulate the cell cycle progression by activating p-Akt, and it’s over expression rescued the inhibition of G1/S transition due to WDHD1 reduction.

G1/S transition is regulated by cyclins, cyclin-dependent kinases (Cdks) and the regulatory proteins [9]. The G1 arrest observed in cells is associated with low levels of G1 Cdks activity and pRb hypophosphorylation. E6 and E7 oncoproteins abrogates cell cycle checkpoints and induces genomic instability by promoting the degradation of the tumour suppressors p53 and pRb, respectively. pRb binds to E2F and inhibits its transcriptional activation of genes, including cyclin A and cyclin E, which are important for the G1-S phase transition. p53 activates its target gene Cdk inhibitor p21 [37], which inactivates the cyclin E1/Cdk2 and cyclin A2/Cdk2 complexes, resulting in pRb hypophosphorylation and cell cycle arrest. Theoretically, p53 expression should be decreased due to E6 and has a low activity during carcinogenesis [38–40]. However, some studies have shown that p53 still functional in E7 expressing cells [41] and can be detected under hypoxia condition or after cytotoxic therapies that cause DNA damage in cervical tumors [42, 43]. These results suggest that p53 is still functioning in cervical cancer cells. Moreover, increased p21 expression was also observed in E7 expressing cells as well as cervical cancer [44, 45] and the expression of both p53 and p21 was increased in low-grade cervical squamous intraepithelial lesions infected with a wide variety of HPV types [46–48]. Furthermore, studies showed that oncogene E6 and E7 expression are different in lower grades of SIL (LSIL), with E7 expression predominating over E6 prior to the development of invasive cervical carcinoma [49]. The above-mentioned illustrated that p53 was still functional in E7 expressing cells as well as cervical cancer.

Polyploid, as one of the manifestations of genomic instability, has been recognized as an important cause of tumor genesis [50]]. The presence of polyploid cells could be examined in the early stages of cervical cancer [51],and our previous study showed that HPV-16 E7 can induce polyploid formation [52]. Generally known, one of the causes of polyploid formation is DNA re-replication, and there are few genes known to induce DNA re-replication currently. Our recent research results showed that the high-risk HPV-16 E7 can induce primary keratinized epithelial cells (PHK) to replicate in the G2 phase by increasing the DNA replication factor Cdt1, resulting in the formation of polyploid cells [20]. We also found that interference with WDHD1 significantly reduced the E7 induced DNA re-replication [16]. Then, whether WDHD1 can induce DNA replication by increasing GCN 5 remains to be further studied.

## Conclusions

In summary, our data elucidate a model that HPV E7 up-regulates the expression of WDHD1 and then GCN5, which activates Akt and thereby promotes cell proliferation. These results will help understand the mechanism by which HPV regulates cell cycle and may contribute to develop drugs against the virus.

## Supplementary information

**Additional file 1.**

**Additional file 2.**

## Data Availability

The data sets used and/or analyzed during the current study available from the corresponding author on reasonable request.

## Abbreviations

WDHD1: WD repeat and high mobility group [HMG]-box DNA-binding protein 1
GCN5: General control nondepressible 5
HAT: Histone acetyltransferase
HPV: Human papillomaviruse
NIKS: Spontaneously immortalized human foreskin keratinocytes
RPE1: Retinal pigment epithelium cell line
BrdU: Bromodeoxyuridine
FACS: Fluorescence-activated cell sorting
CHX: Cycloheximide
PI3K: Phosphoinositide 3-kinase
