# Supplementary material for: WDHD1 facilitates G1 checkpoint abrogation in HPV E7 expressing cells by modulating GCN5
Source: BMC Cancer. 2020 Sep 3;20:840. doi: 10.1186/s12885-020-07287-1 (PMC7469104; doi:10.1186/s12885-020-07287-1)
Supplement: Supplementary file 1 — Additional file 1. [file 12885_2020_7287_MOESM1_ESM.docx]

**Supplementary methods**

**Cell culture.** Spontaneously immortalized human foreskin keratinocytes (NIKS cells) were cultured on mitomycin C-treated J2-3T3 feeder cells with E medium, which was composed by 1 part Ham’s F12 medium plus 5% fetal bovine serum (FBS) and 3 parts Dulbecco’s modified Eagle medium (DMEM). Cells of the human telomerase reverse transcriptase-expressing human retinal pigment epithelium cell line RPE1 were maintained in a 1:1 dilution of DMEM-Ham’s F-12 medium plus 10% FBS. pBabe retroviral system was used to establish the HPV-16 E7 expressing NIKS and RPE1 cells as described previously[[1](#_ENREF_1)]. Puromycin was used to maintain the above two cell lines, which are limited to be used within 15 generations[[2](#_ENREF_2)].

**RNA-seq.** Total RNA from NIKS cells was used to construct cDNA libraries for RNA-seq[[2](#_ENREF_2)]. First, the rRNA was removed by the use of a Ribo-Minus kit (Life Technologies) combined with custom-designed DNA probes for rRNAs. The processed total RNA was then used to construct RNA-seq libraries with a NEBNext mRNA library preparation kit (New England BioLabs). In this way, double-stranded cDNA was synthesized from rRNA-depleted total RNA. RNA was then end repaired, dA tailed, and ligated to standard Illumina adaptor oligonucleotides. Adaptor-ligated cDNA libraries were amplified with Phusion PCR master mix. The cDNA libraries were then loaded into a HiSeq 2000 system (Illumina) for sequencing at the Washington University Genome Technology Access Center. The resulting raw sequence reads were first analyzed using a custom bioinformatics pipeline to remove low-quality reads and then clustered before sequentially mapping to the human transcriptome and genome with Bowtie was performed. In this way, about 90% of all sequence reads were mapped to known human sequences. Sequence reads mapping to the same transcript were combined and normalized on the basis of the length of the transcript and the number of total reads from each sample (reads per kilobase per million [RPKM]). Normalized read counts were compared across samples to identify changes in transcript abundance. Transcripts from the same gene locus in the human genome were combined for evaluation of expression changes at the gene level.

**Flow cytometry.** For cell cycle and polyploidy analysis, asynchronous cultured cells expressing HPV E7 or vector alone was treated with phosphate buffered saline (PBS) or bleomycin (Alexis Biochemicals) (4μg /ml in PBS). At 24 h later, cells were fixed in 70% ethanol, treated with 50 μg /ml RNase A plus 50μg /ml propidium iodide (PI), and analyzed by fluorescence activated cell sorting (FACS). For the bromodeoxyuridine (BrdU) labeling experiment, BrdU (final concentration, 20μM) was added to the medium 2 h before collection of cells. After fixation, cells were permeabilized with 2 N HCl–0.5% Triton X-100, neutralized with 0.1 M sodium tetraborate, stained with monoclonal anti-BrdU (BD Biosciences) followed by treatment with anti-mouse IgG F(ab)2-fluorescein isothiocyanate (FITC) (Sigma), and counterstained with PBS–7-aminoactinomycin D (7-AAD)–RNase A. Flow cytometric analysis was performed on a BD FACSAria III sorter instrument equipped with BD FACSDiva 7.0 software (BD Biosciences, NJ, USA). FITC 490-nm fluorescence was acquired in logarithmic amplification in FL1, and 7-AAD 650-nm fluorescence was acquired in linear amplification in FL3. Cell cycle analysis was done using a Cytomics FC500 Flow Cytometry CXP 2.0 system. The detailed experimental steps need to follow the published articles[[2](#_ENREF_2)].

**siRNAs and transfection.** The Invitrogen Lipofectamine 2000 transfection reagent was used for gene knockdown (with a final concentration of 20 nM small interfering RNA (siRNA) per target gene) and cell cycle analysis in E7 and vector-control expressing cells**.** For gene knockdown analysis, cells were harvested 48 h post transfection and specific protein levels were analyzed by immunoblotting. For cell cycle analysis, 24 h after transfection, cells were treated with bleomycin (4 μg/ml) for an additional 36 h. The sequence of siRNA duplexes were in table 1. Detailed experimental steps need to follow our published article[[2](#_ENREF_2)].

**References**

1. Fan X, Liu Y, Heilman SA, Chen JJ: **Human papillomavirus E7 induces rereplication in response to DNA damage**. *Journal of virology* 2013, **87**(2):1200-1210.

2. Zhou Y, Zhang Q, Gao G, Zhang X, Liu Y, Yuan S, Wang X, Chen JJ: **Role of WDHD1 in Human Papillomavirus-Mediated Oncogenesis Identified by Transcriptional Profiling of E7-Expressing Cells**. *Journal of virology* 2016, **90**(13):6071-6084.
