# Supplementary figures and images for: WDHD1 facilitates G1 checkpoint abrogation in HPV E7 expressing cells by modulating GCN5
Source: BMC Cancer. 2020 Sep 3;20:840. doi: 10.1186/s12885-020-07287-1 (PMC7469104; doi:10.1186/s12885-020-07287-1)

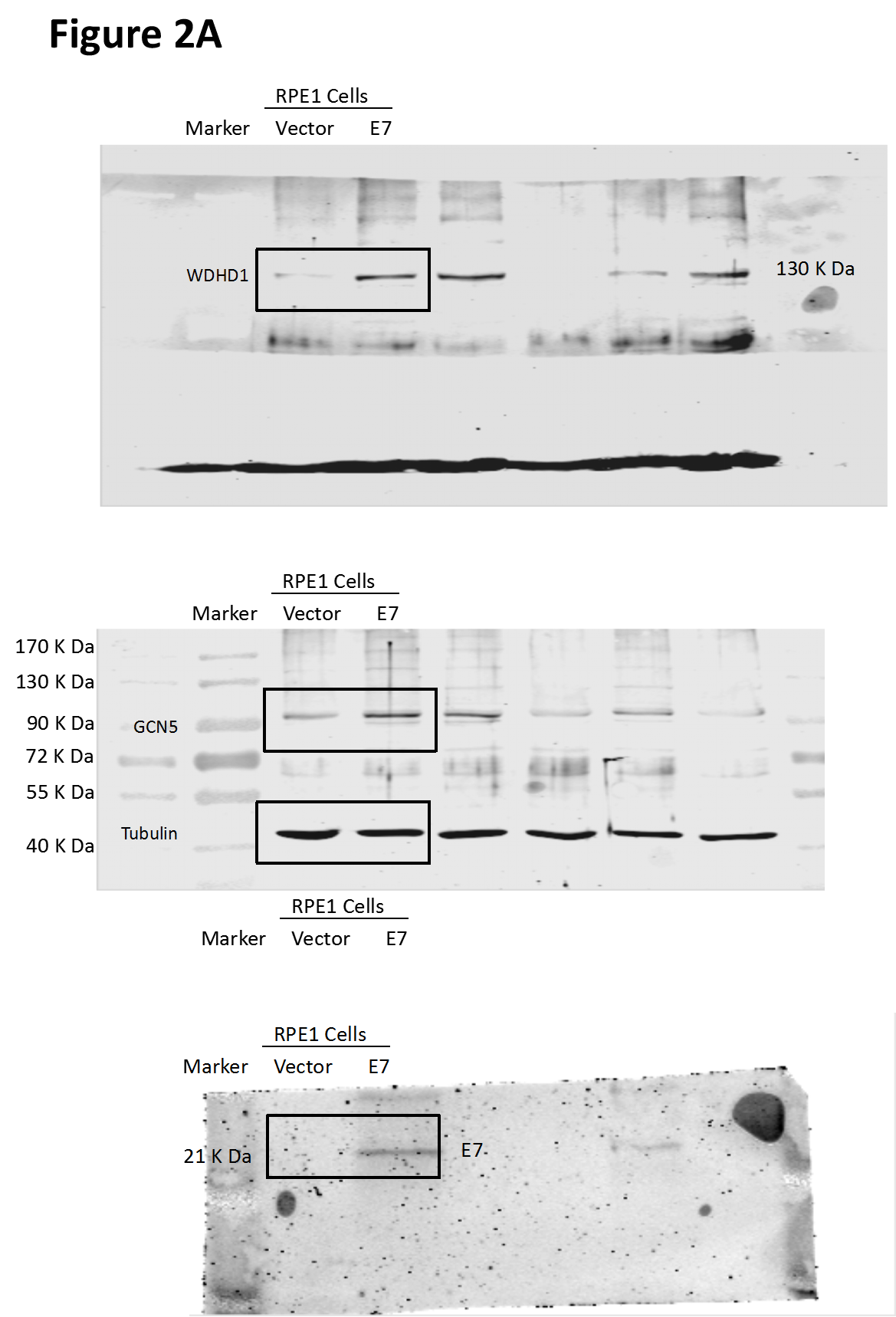


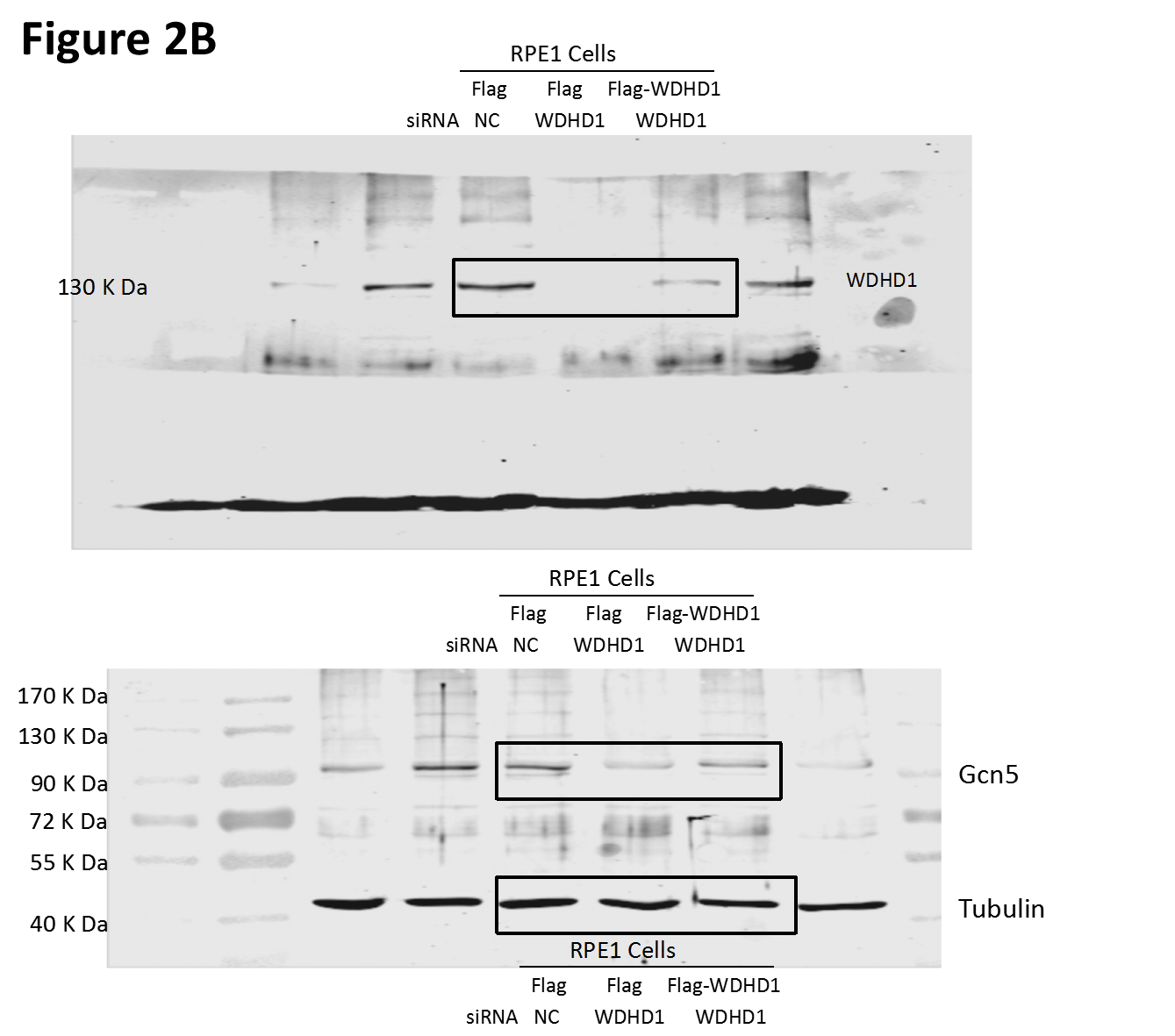


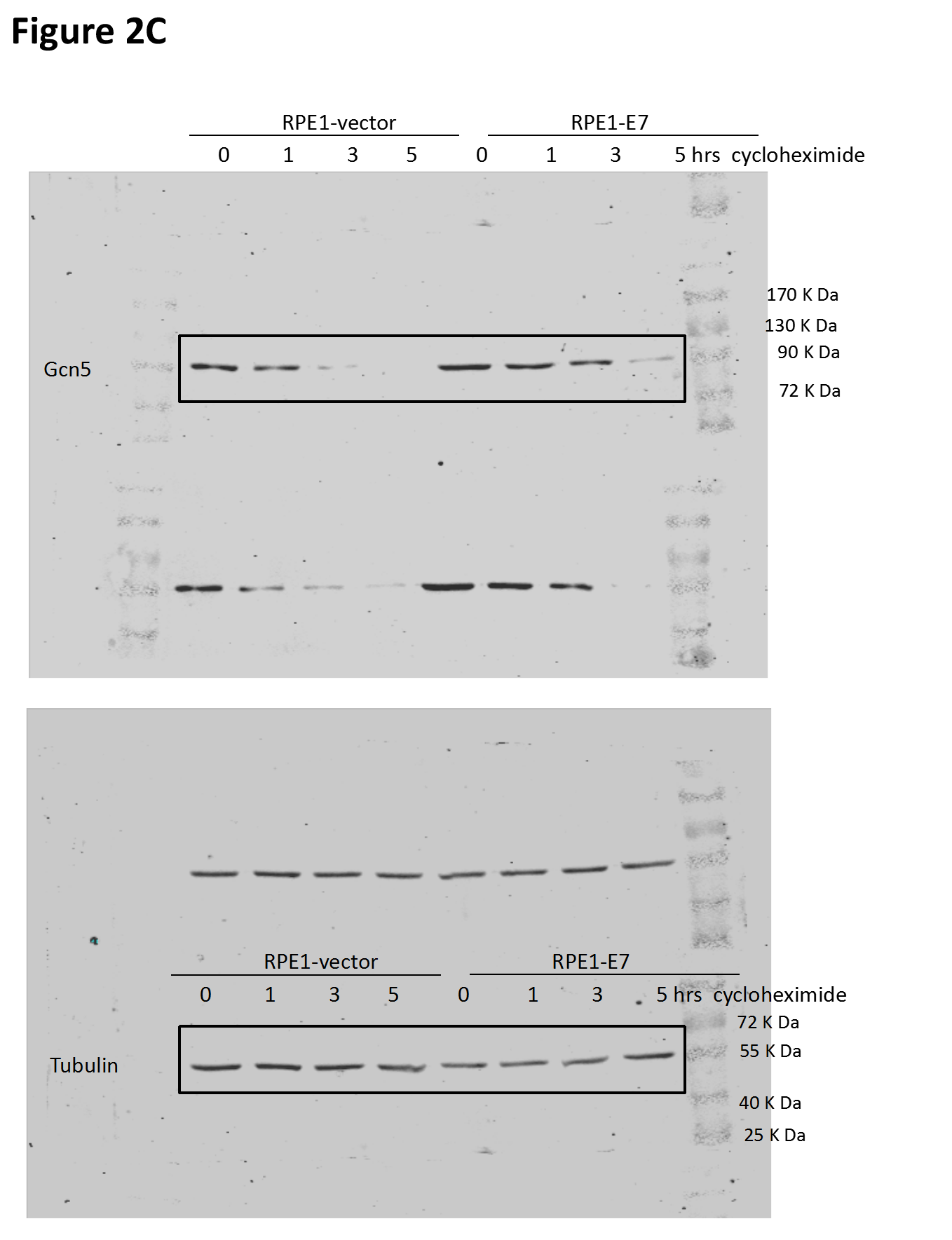


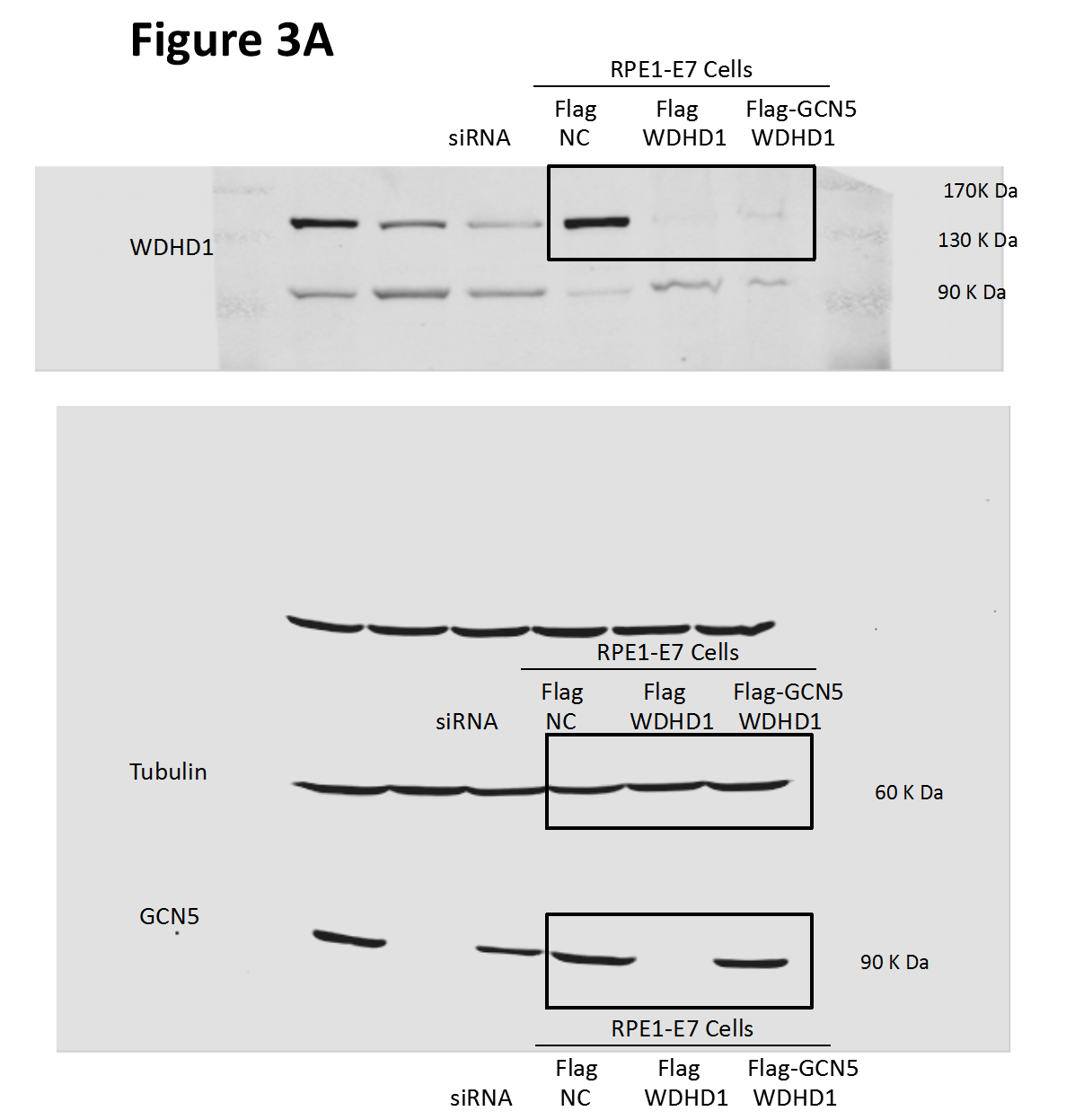


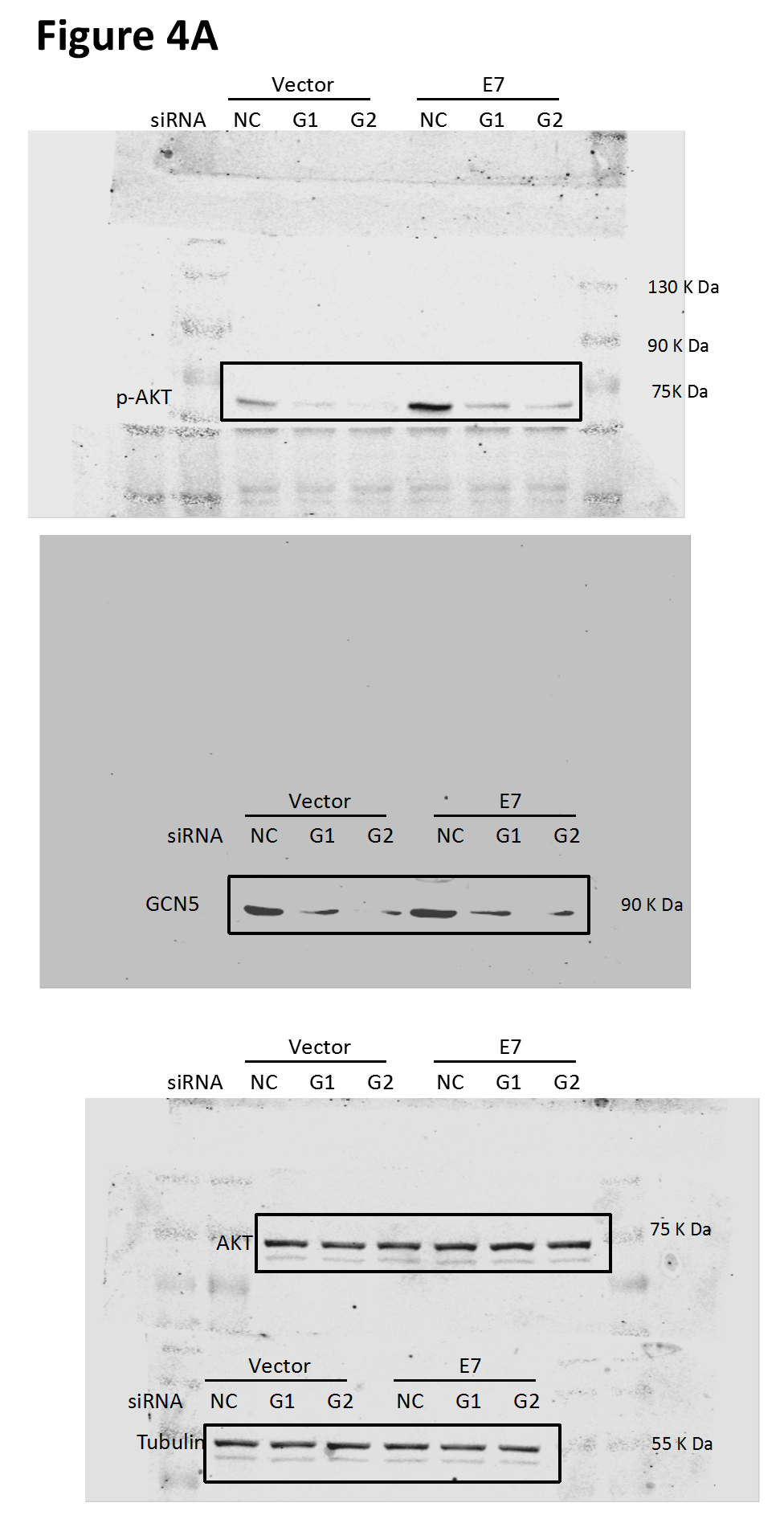


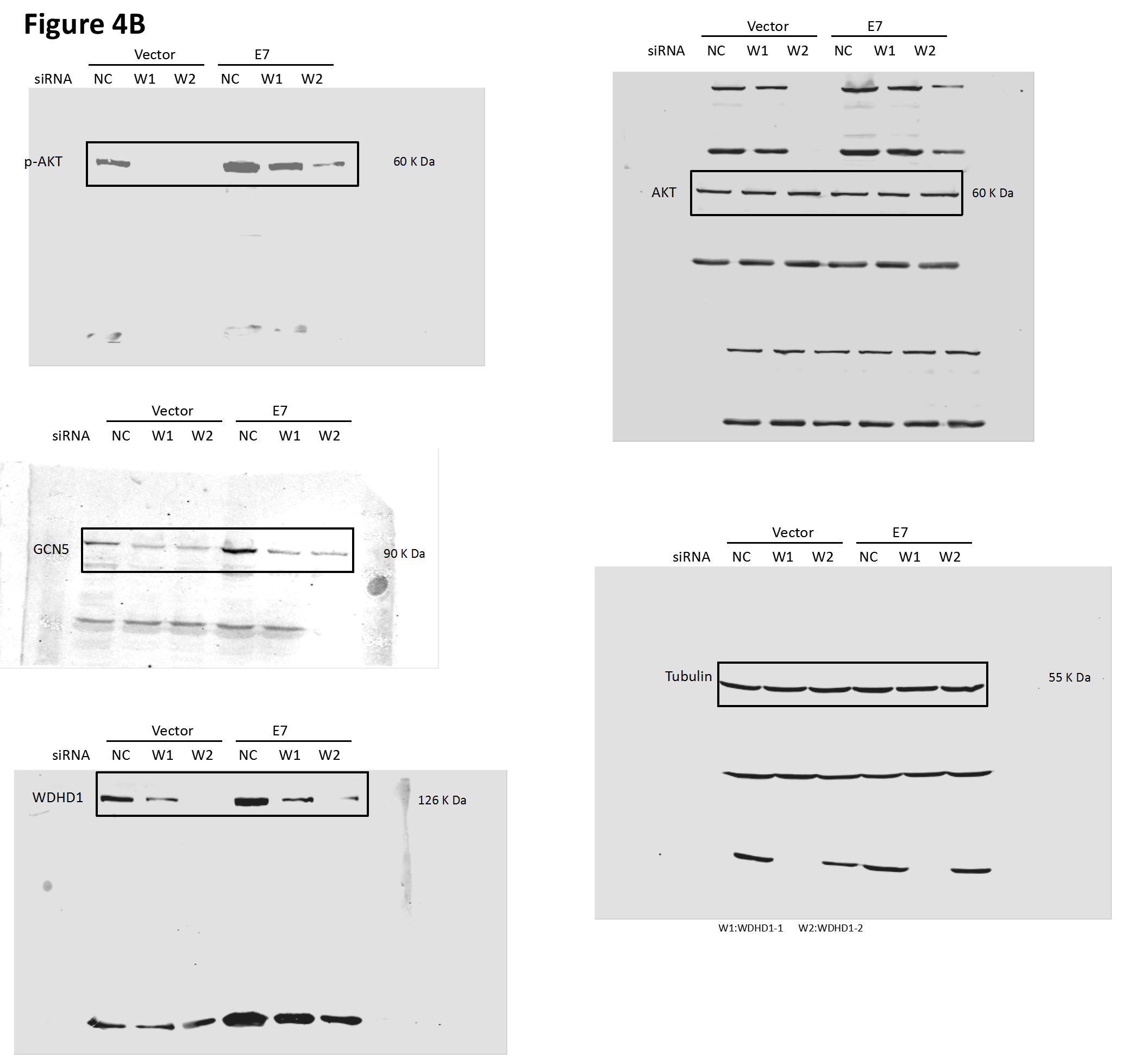


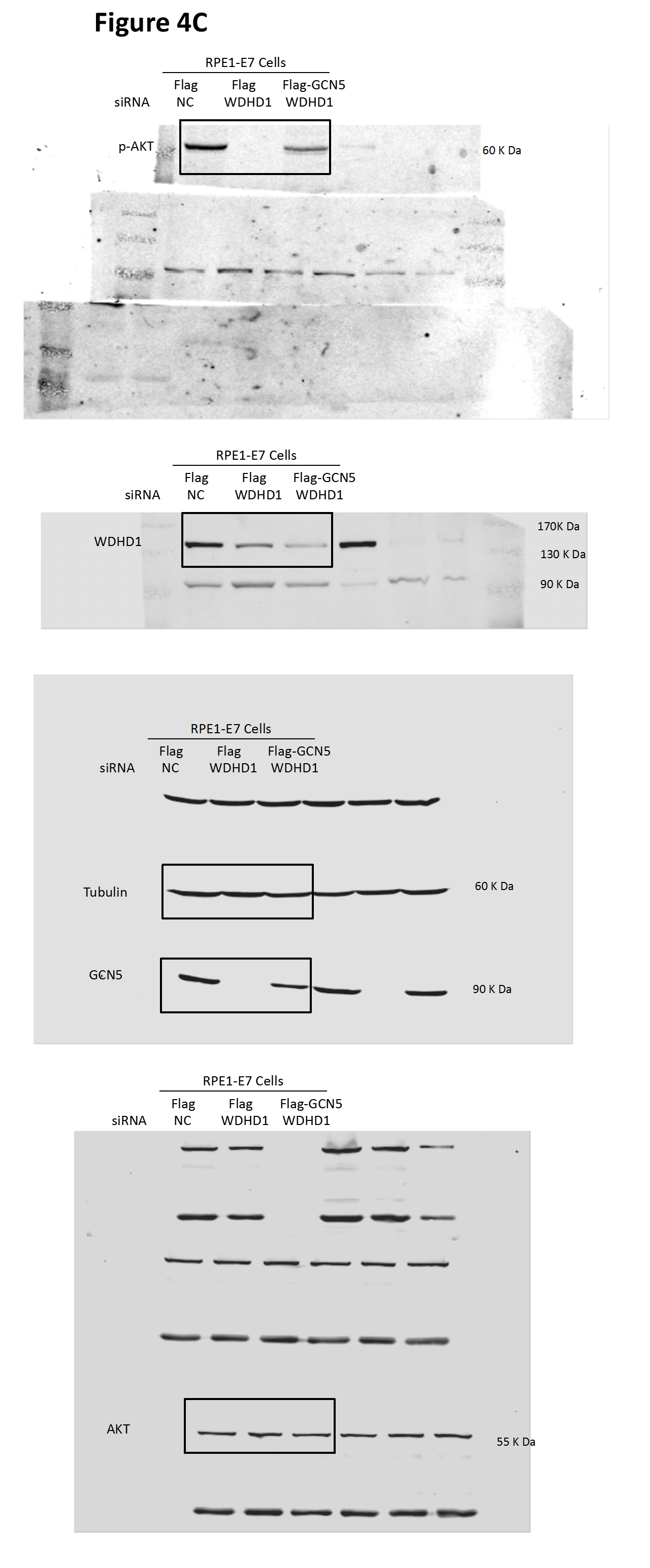

Supplement: Supplementary file 2 — Additional file 2. [file 12885_2020_7287_MOESM2_ESM.doc]
